# Association between gynecological disorders and insomnia and depression trajectories: a longitudinal study of middle-aged women

**DOI:** 10.3389/fpsyt.2024.1515678

**Published:** 2025-01-07

**Authors:** Huiyong Zhong, Fuling Zeng

**Affiliations:** Department of Gynaecology, Guang Zhou Baiyun District Maternal and Child Health Hospital, Guangzhou, China

**Keywords:** pelvic pain, pelvic prolapse/relaxation, abnormal bleeding, insomnia, depression

## Abstract

**Background:**

Insomnia and depression often receive inadequate attention regarding their association with common menopausal gynecological disorders (GDs), and there is a lack of longitudinal epidemiological evidence. Furthermore, the specific disorders that exhibit the strongest correlation with depression, as well as the potential mediating role of insomnia, remain poorly understood.

**Methods:**

Using data from the Study of Women’s Health Across the Nation (SWAN) spanning 1996 to 2008, this study analyzed a sample of 2217 racially diverse premenopausal women (aged 42 to 53 at baseline). Longitudinal trajectory analysis, employing latent class mixture models (LCMM), was used to identify optimal patterns for insomnia and depression. Logistic regression explored associations between pelvic pain, pelvic prolapse or relaxation, abnormal bleeding, and insomnia/depression trajectories. A causal mediation model investigated whether insomnia mediated the link between gynecological disorders and depression.

**Results:**

The analysis included 2217 participants for insomnia trajectories and 1767 for depression trajectories. Insomnia and depression showed similar patterns, with a single high and low trajectory and minimal fluctuations. Logistic regression revealed a significant positive correlation between pelvic pain, abnormal bleeding, number of GDs, and an increased risk of high insomnia and depression trajectories. Insomnia trajectories mediated 23.6%, 14.3%, and 11.9% of the association between pelvic pain, abnormal bleeding, and number of GDs, respectively, with depression trajectories.

**Conclusions:**

This study found the significant associations between pelvic pain, abnormal bleeding, and comorbidity with an elevated risk of insomnia and depression during the menopausal transition.

## 1 Introduction

Depression, a prevalent mental disorder, is characterized by prolonged emotional suffering or a loss of interest or pleasure in activities (1). It poses a significant burden worldwide, affecting approximately 300 million people (2). Notably, women exhibit a higher susceptibility to depression than men, with a twofold increased likelihood according to research conducted in developed countries (3, 4). This gender disparity emerges during adolescence and persists until around the age of 50 (5). Two critical stages of reproductive health, namely pregnancy and the menopausal transition, are closely linked to the development of depression. Notably, a study by E. W. Freeman et al. (6) found that women are 2.5 times more susceptible to depression during the menopausal transition compared to the premenopausal phase. Recently, a related systematic review and meta-analysis has strengthened this evidence, highlighting the vulnerability of women’s mental health during the menopausal transition (7).

When examining the contribution of unique factors to depression among middle-aged women, common gynecological disorders such as pelvic pain, pelvic prolapse/relaxation, and abnormal bleeding emerge as significant but incompletely understood factors. For instance, the impact of abnormal bleeding resulting from hormonal fluctuations during menopause often goes unnoticed, downplaying its influence on depression (8). Notably, studies conducted in young and middle-aged women have produced conflicting findings on this topic (9, 10). Moreover, although recent research has identified a positive association between pelvic pain and the worsening of depressive symptoms in women (11), it is essential to acknowledge the limitations of its cross-sectional design, restricted sample size, and the absence of evidence from longitudinal studies.

Sleep disorder and insomnia, particularly during the menopausal transition, present significant health challenges for women (12–14). In a study by Fiona C. Baker et al. (15), sleep diaries and polysomnography were employed to reveal the contribution of objective physiological hot flashes to sleep disturbances in women undergoing the menopausal transition. This finding suggests a potential link between the physiological health status during the menopausal transition and the development of sleep disruption. In particular, a randomized controlled trial by Sandlund et al. (16) of insomnia treatment in patients with an average age of 54 years, the vast majority of whom were women, showed that improvement in insomnia significantly reduced depressive symptoms. However, current evidence is largely limited to cross-sectional surveys or case-control studies, which may not adequately clarify the underlying causal relationships. Given the limited longitudinal evidence on the independent contributions of GDs to sleep disorders and depression, and the uncertainty regarding whether sleep disorders may mediate the relationship between GDs and depression, there is a need for further investigation in long-term cohort studies.

In recent years, an increasing number of statistical methods for longitudinal studies have been developed, including latent class mixture models (LCMM). LCMM is a powerful tool for identifying distinct subgroups within a population based on longitudinal data (17). Unlike traditional methods that assume a homogeneous population, LCMM captures the heterogeneity in the data by modeling individual trajectories over time. This method allows researchers to detect unobserved subgroups that may follow different patterns of change, offering more nuanced insights into the underlying processes. In longitudinal research, LCMM accounts for both within-subject and between-subject variability, making it particularly useful for understanding complex relationships.

Therefore, this study aims to use LCMM to investigate whether GDs have a direct effect on both insomnia and depression, and whether insomnia acts as a mediator between GDs and depression. We hypothesize that (1) GDs directly affect insomnia and depression, and (2) insomnia mediates the relationship between GDs and depression. These hypotheses will be tested using a longitudinal approach.

## 2 Materials and methods

### 2.1 Study population

This study utilized publicly available data from the nationally representative Study of Women’s Health Across the Nation (SWAN) datasets. SWAN is a longitudinal, multicenter study conducted within the community, focusing on the natural processes of the menopausal transition and aging in middle-aged women (18). The study recruited 3,302 women aged 42 to 53 years from 1996 to 1997, encompassing 7 geographically diverse sites in the United States. All participants in SWAN were between the ages of 42 and 52, had a complete uterus, had experienced at least one menstrual period, had not used reproductive hormones in the previous 3 months, and did not have a history of total hysterectomy at baseline. Following completion of the baseline survey, SWAN participants in the public dataset were annually followed up. The research protocol received approval from the institutional review board at each study site.

**Figure 1:**
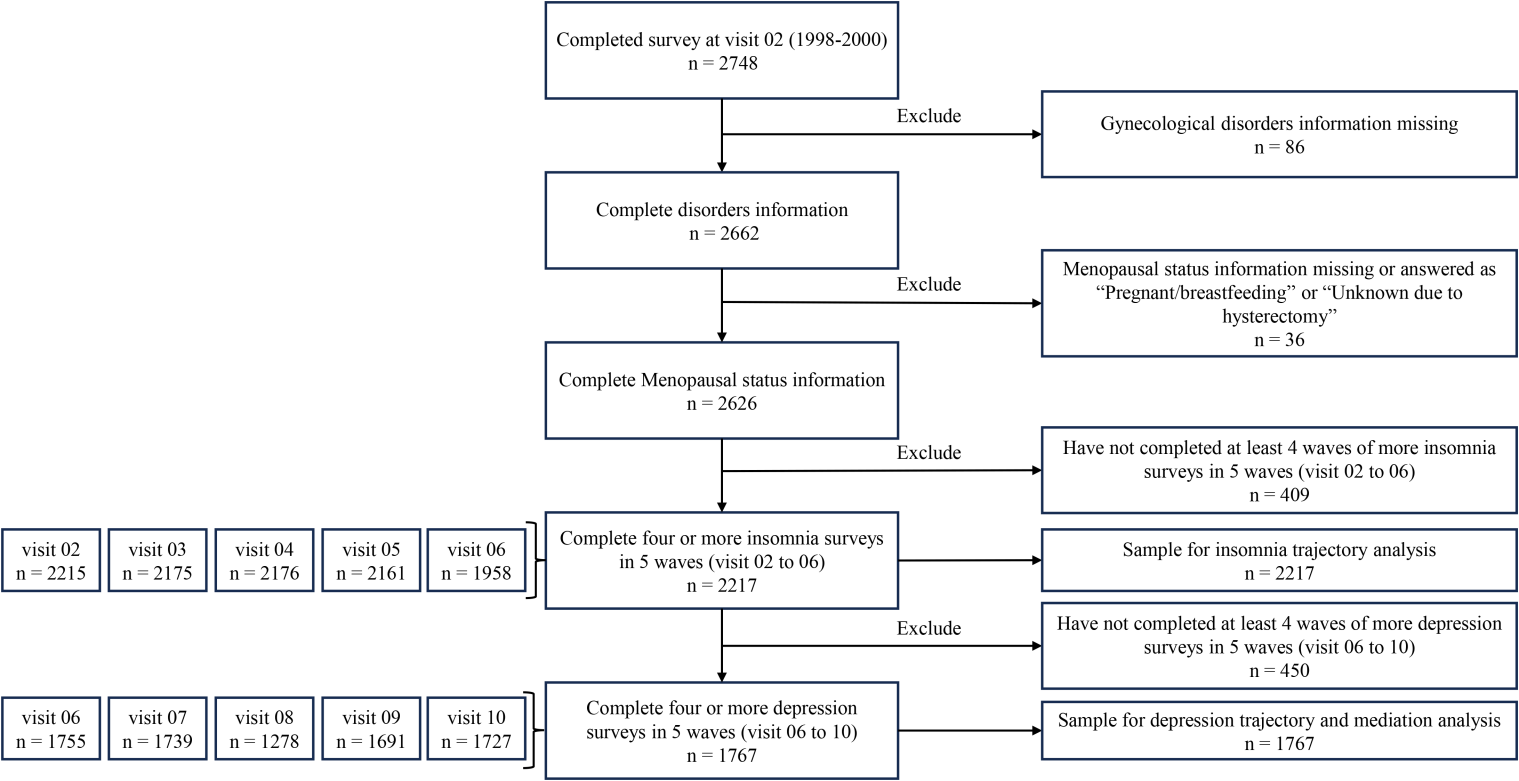
Flowchart of population used for analysis.

During the second follow-up period (1998-2000), SWAN incorporated a survey on gynecological disorders in women, which included pelvic pain, pelvic prolapse, and abnormal bleeding. For this study, we selected 2,748 participants who had completed the second follow-up survey. Initially, 86 subjects were excluded due to missing information on the aforementioned three conditions. We then excluded subjects with missing information on menopausal status, those who responded during pregnancy or lactation, and those who had undergone hysterectomy, for a total of 36 subjects. Furthermore, we excluded 409 participants who had not completed at least four insomnia questionnaires between the second and sixth follow-up visits. The remaining 2217 participants were included in the analysis of insomnia trajectories. We then excluded 450 participants who did not complete at least four depression surveys between the sixth and tenth follow-ups, and based on these data (1767 participants) we conducted depression trajectory analyses and mediation analyses. **Figure 1** illustrates the flowchart depicting the analyzed population.

### 2.2 Gynecological disorders assessment

The primary exposure of interest in this study was GDs reported during the second follow-up period. Participants were asked by SWAN whether they experienced pelvic pain, pelvic prolapse/relaxation, or abnormal bleeding since their last visit. If participants replied “yes”, they were regarded as suffering from the corresponding GD.

### 2.3 Insomnia symptoms assessment

During each follow-up, participants reported the frequency of three sleep-related habits over the past two weeks: difficulty falling asleep, waking up multiple times during the night, and waking up earlier than planned and being unable to fall asleep again. Response options ranged from “none in the past two weeks” to “5 or more times a week.” Based on previous SWAN publications (19), each option in this study was scored from 0 to 4, resulting in a total score ranging from 0 to 12. A higher score indicated more severe insomnia symptoms.

### 2.4 Depression symptom assessment

At each annual follow-up visit, well-trained assessors used the 20-item Center for Epidemiological Studies Depression Scale (CES-D) to evaluate the participants’ depressive symptoms over the past week (20). Respondents rated each item on a standardized Likert scale ranging from 0 = never to 3 = most or all of the time. The total CES-D score ranged from 0 to 60, with higher scores indicating more severe depression. A cut-off points of 16 or higher was considered clinically significant for depressive symptoms.

### 2.5 Covariates

The selected covariates included age (years old), BMI (kg/m^2^), race (Black/African American, Chinese/Chinese American, Japanese/Japanese American, Caucasian/White non-Hispanic, and Hispanic), marital status (single/never married, currently married or living as married, separated, widowed, and divorced), menopausal status (post by bilateral salpingo oophorectomy, natural post, late perimenopause, early perimenopause, premenopausal, and unknown due to hormones use), education (less than high school, high school only, some college/technical school, college degree, and post graduate education), annual family income (less than $19, 999, $20, 000 to $49, 999, $50, 000 to $99, 999, and $100,000 to $ more), smoking (no and yes), and history of depression (no and yes). Considering that a history of hormone replacement therapy (HRT) could be a potential confounder in the association between GDs and insomnia and depression (21), we accounted for this factor in our sensitivity analyses. A subject was considered to have a history of hormone replacement therapy if they had used any of the following: Estrogen pills (such as Premarin, Estrace, Ogen, etc.), Estrogen by injection or patch (such as Estraderm), Combination estrogen/progestin (such as Premphase or Prempro), or Progestin pills (such as Provera). Age information was obtained from the baseline survey, while the history of depression was determined by whether the CES-D score was 16 or higher at baseline and the first follow-up. Data on the remaining variables were collected during the second survey wave.

### 2.6 Statistical analysis

#### 2.6.1 Latent class mixture model

A latent class mixture model (LCMM) employing maximum likelihood parameter estimation was used to identify group-based longitudinal trajectories for repeated measures of insomnia and depression (17). The fit of LCMM models with one to five trajectory classes was examined using linear functions. The optimal trajectory class model was determined based on the following criteria: (a) Akaike information criterion (AIC) and Bayesian information criterion (BIC), with smaller values indicating better model fit; (b) Ensuring that each trajectory had at least 10% of the total sample size; (c) Requiring a mean posterior probability greater than 80% for each trajectory class; (d) Relative entropy values greater than 0.5; (e) Considering the interpretability and research significance of the identified trajectories. To mitigate the risk of local maxima, an automatic grid search with 30 random initial values was employed to fit the LCMM.

#### 2.6.2 Logistic regression

The identified insomnia and depression trajectories served as the dependent variables for regression analysis, with their respective low-level trajectories selected as the reference group. Binary or multinomial logistic regression models were used to explore the association between GDs and insomnia and depression trajectories, respectively. The effects were represented by odds ratios (*OR*) and 95% confidence intervals (*CI*). Two models were tested: a crude model without adjusting for any covariates, and an adjusted model that included age, BMI, race, marital status, menopausal status, education, family income, smoking, and depression history. Unadjusted and adjusted regression models were also tested for the association between insomnia and depression trajectories. Missing covariate information is presented in **Supplementary Table S1**, and multivariate interpolation was conducted using the chain equation.

### 2.6.3 Causal mediation analysis

Model-based causal mediation analysis (22) was conducted using the Mediation R package to examine whether the exposure (GDs) was indirectly associated with the outcome (depression) through the mediator (insomnia). This mediation analysis approach is based on the counterfactual framework of causal inference and does not rely on any specific statistical model. We defined *M_i_ (e)* as the potential value of the mediator of interest for participant *i* under exposure state e, and *Y_i_ (e, m)* as the possible outcomes of exposure at level e and mediation at level m. Assuming that both the exposure and mediation states were binary (0 and 1), the total effect could be represented by the following equation:


$$\tau_{i}=E\left(Y_{i}\left(1,\;M_{i}\left(1\right)\right)-Y_{i}\left(0,\;M_{i}\left(0\right)\right)\right);$$

The causal mediation effect (indirect effect) could be described as:


$$\delta_{i}\left(e\right)=E\left(Y_{i}\left(e,\;M_{i}\left(1\right)\right)-Y_{i}\left(e,\;M_{i}\left(0\right)\right)\right);$$

Furthermore, the directed effect could be represented by:


$$\zeta_{i}\left(e\right)=E\left(Y_{i}\left(1,\;M_{i}\left(m\right)\right)-Y_{i}\left(0,\;M_{i}\left(m\right)\right)\right).$$

The ACME (average causal mediation effect) and ADE (average direct effect) were estimates of *δ_i_ (e)* and *ζ_i_ (e)*, respectively. To calculate the ACMEs of insomnia in this study, we fitted the mediator model for the probability of insomnia trajectories, including the single GD and all covariates as independent variables, and the outcome model for the probability of depression trajectories, including the single GD, insomnia trajectories, and all covariates as independent variables. All analyses were performed using R 4.2.2, with statistical significance set at *P*<0.05.

#### 2.6.4 Sensitivity analysis

We conducted several sensitivity analyses to validate our results. First, in the LCMM analysis, we performed two analyses: one included only subjects who completed five assessments of insomnia and depression; the second used the inverse probability weighting (IPW) method. Second, in the logistic regression analysis, we performed the same two analyses as above and additionally included a history of hormone replacement therapy as a potential confounder in the model. Third, robust standard errors were calculated and applied to a quasi-Bayesian simulation in the causal mediation analysis. Lastly, we compared the basic characteristics of included and excluded subjects to explain potential selection bias.

## 3 Results

### 3.1 Descriptive statistics

Among the final sample of 1767 individuals, the average age of the subjects was 45.93 ± 2.642 years, with an average BMI of 27.92 ± 7.214 kg/m². Of the participants, 51.6% were Caucasian, 68.6% were married, 56.9% were in early perimenopause, 50.2% had a college degree or higher, 26.2% had a history of depression, and 11.5% had a history of hormone replacement therapy. Among the subjects, 15.1%, 2.7%, and 15.4% had pelvic pain, pelvic prolapse/relaxation, and abnormal bleeding, respectively. Additionally, 5.8% of women had two or more of these gynecological disorders.

### 3.2 Group-based trajectories of insomnia and depression

**Figure 2:**
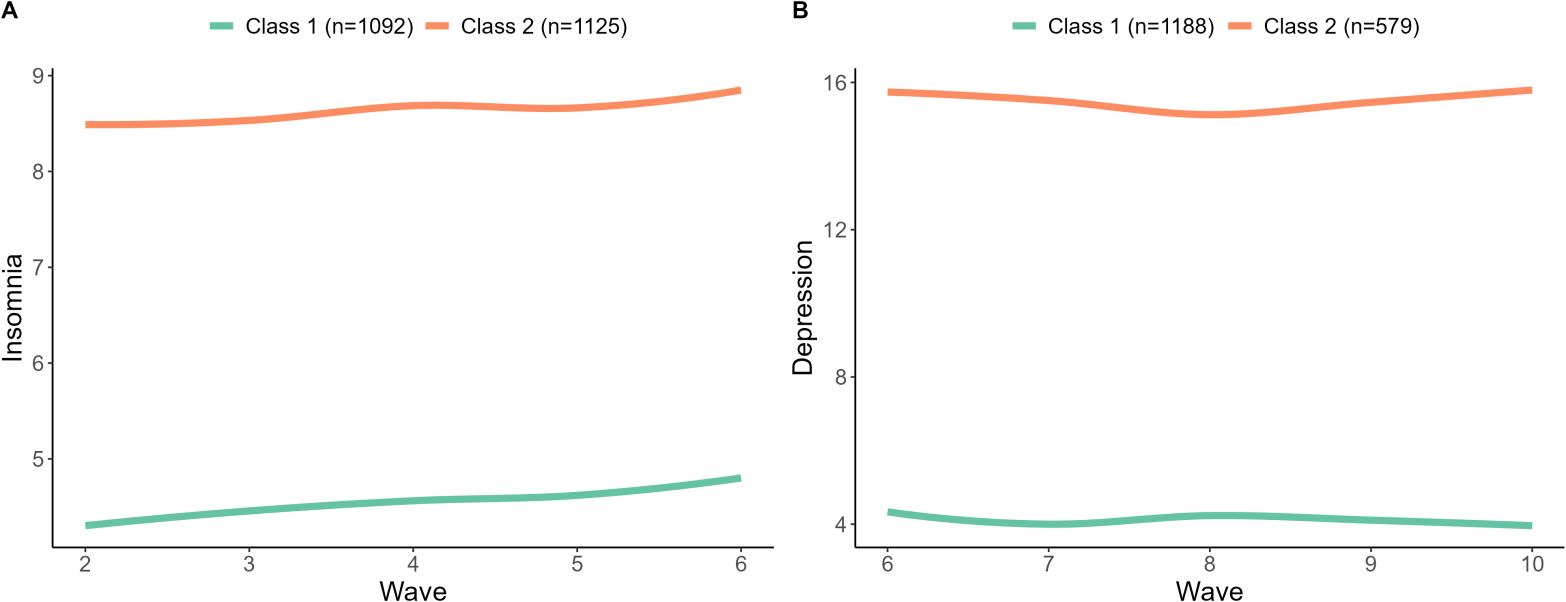
Group-based trajectories of insomnia **(A)** and depression **(B)**. All participants were classified into two trajectory categories. The trajectory categories represented different patterns of insomnia **(A)** and depression **(B)** over time. The mean posterior probabilities for these categories were greater than 80%.

We identified group-based trajectories for both insomnia and major depression by LCMM. Separate models were fitted for each outcome based on different populations. The full fit indices for the 1- to 5-class models are presented in **Supplementary Table S2**. The optimal trajectories representing the longitudinal patterns of insomnia and major depression are shown in **Figure 2**. The trajectories for insomnia and depression exhibit similar patterns, characterized by a high and a low trend with minimal fluctuations. Hence, we referred to these trajectories as the ‘low insomnia’ and ‘high insomnia’ trajectory groups for insomnia, and the ‘low depression’ and ‘high depression’ trajectory groups for depression.

We further compared the distribution of basic characteristics and GDs across different trajectories of insomnia and depression. The results revealed significant differences in BMI, race, annual family income, smoking, history of depression, pelvic pain, abnormal bleeding, and the number of GDs between different insomnia trajectories (**Supplementary Table S3**) and depression trajectories (**Supplementary Table S4**).

### 3.3 Association between gynecological disorders and insomnia

The logistic regression analysis results, presented in **Table 1**, indicated significant associations between GDs and insomnia trajectories. Specifically, pelvic pain (adjusted *OR* = 1.88, 95%*CI* = 1.46-2.42) and abnormal bleeding (adjusted *OR* = 1.41, 95%*CI* = 1.10-1.81) are associated with high-level insomnia symptoms. However, there is no association between high-level insomnia trajectories and pelvic prolapse/relaxation. Additionally, having one GD (adjusted *OR* = 1.49, 95%*CI* = 1.20-1.86) or two or more GDs (adjusted *OR* = 2.08, 95%*CI* = 1.38-3.12) significantly increases the risk of high insomnia symptoms compared to the population without any GD.

**Table 1:** Association between gynecological disorders and insomnia trajectories.

Gynecological disorders	Crude model		Adjusted Model	
	*OR* (95% *CI*)	*P*	*OR* (95% *CI*)	*P*
Pelvic pain				
No	1.00 (Ref.)		1.00 (Ref.)	
Yes	2.07 (1.63-2.64)	<0.001	1.88 (1.46-2.42)	<0.001
Pelvic prolapse or relaxation				
No	1.00 (Ref.)		1.00 (Ref.)	
Yes	1.45 (0.87-2.43)	0.156	1.17 (0.68-2.01)	0.574
Abnormal bleeding				
No	1.00 (Ref.)		1.00 (Ref.)	
Yes	1.63 (1.28-2.06)	<0.001	1.41 (1.10-1.81)	0.008
Number of GDs				
0	1.00 (Ref.)		1.00 (Ref.)	
1	1.56 (1.27-1.92)	<0.001	1.49 (1.20-1.86)	<0.001
≥2	2.72 (1.85-4.00)	<0.001	2.08 (1.38-3.12)	<0.001
*P* for trend	–	<0.001	–	<0.001

“Low” as reference trajectory; Crude model: no adjustment; Adjusted model: adjusted for age, BMI, race, marital status, menopausal status, education, family income, smoking, and depression history.

### 3.4 Association between gynecological disorders and depression

After adjusting for covariates, pelvic pain (adjusted *OR* = 1.47, 95%*CI* = 1.09-1.98), pelvic prolapse or relaxation (adjusted *OR* = 1.93, 95%*CI* = 1.01-3.71), abnormal bleeding (adjusted *OR* = 1.48, 95%*CI* = 1.09-2.01), and the number of GDs (having one GD: adjusted *OR* = 1.51, 95%*CI* = 1.15-1.98; having two or more GDs: adjusted *OR* = 1.80, 95%*CI* = 1.13-2.85) are positively associated with high-level depressive trajectories (**Table 2**).

**Table 2:** Association between gynecological disorders and depression trajectories.

Gynecological disorders	Crude model		Adjusted Model	
	*OR* (95%CI)	*P*	*OR* (95%CI)	*P*
Pelvic pain				
No	1.00 (Ref.)		1.00 (Ref.)	
Yes	1.70 (1.30-2.22)	<0.001	1.47 (1.09-1.98)	0.011
Pelvic prolapse or relaxation				
No	1.00 (Ref.)		1.00 (Ref.)	
Yes	2.10 (1.18-3.73)	0.012	1.93 (1.01-3.71)	0.049
Abnormal bleeding				
No	1.00 (Ref.)		1.00 (Ref.)	
Yes	1.51 (1.16-1.97)	0.002	1.48 (1.09-2.01)	0.012
Number of GDs				
0	1.00 (Ref.)		1.00 (Ref.)	
1	1.41 (1.11-1.79)	0.006	1.51 (1.15-1.98)	0.003
≥2	2.33 (1.55-3.50)	<0.001	1.80 (1.13-2.85)	0.013
*P* for trend	–	<0.001	–	<0.001

“Low” as reference trajectory; Crude model: no adjustment; Adjusted model: adjusted for age, BMI, race, marital status, menopausal status, education, family income, smoking, and depression history.

### 3.5 Association between insomnia and depression

The “low” trajectory of both insomnia and depression served as the reference group in the association between insomnia and depression. The results indicated a significant association between high levels of insomnia and an increased risk of severe depression trajectories (crude *OR* = 2.75, 95%*CI* = 2.23-3.38, *P* < 0.001). After adjusting for covariates, this association remains significant (adjusted *OR* = 2.49, 95%*CI* = 1.97-3.14, *P* < 0.001).

### 3.6 Mediating effect of insomnia

Causal mediation analyses were conducted separately for pelvic pain, abnormal bleeding, and the number of disorders. The results indicate that insomnia mediates 23.6%, 14.3%, and 11.9% of the total effect between pelvic pain, abnormal bleeding, the number of GDs, and depression, respectively (**Table 3**).

**Table 3:** The mediating effect of insomnia in the association between gynecological disorders and depression trajectories.

Gynecological disorders	ACME *OR* (95% *CI*)	ADE *OR* (95% *CI*)	Total effect *OR* (95% *CI*)	PME (%)
Pelvic pain	1.018 (1.006-1.031)	1.057 (1.002-1.116)	1.076 (1.015-1.139)	23.6
Abnormal bleeding	1.010 (1.000-1.020)	1.067 (1.007-1.139)	1.078 (1.015-1.150)	14.3
Number of GDs	1.010 (1.001-1.020)	1.073 (1.018-1.128)	1.084 (1.028-1.139)	11.9

ACME, average causal mediation effect; ADE, average direct effect; PME, proportion of mediated effect.

### 3.7 Sensitivity analysis

**Supplementary Tables S5**-**S8** present the results of the analyses for subjects who completed the five-wave survey and those using inverse probability weighting, respectively. The results are consistent with the main analysis, demonstrating that pelvic pain, abnormal bleeding, and the number of GDs increase the risk of high-level insomnia and depression trajectories. After additionally accounting for the history of hormone replacement therapy as a confounder, only the association between pelvic prolapse or relaxation and depression became marginally significant, while all other results remained consistent with the primary analysis (**Supplementary Table S9**). Robust standard errors were calculated and applied to quasi-Bayesian simulations in the analysis of causal mediators. The results of this analysis are essentially consistent with the main analysis (**Supplementary Table S10**). However, the results in **Supplementary Table S11** showed that there were significant differences in the distribution of basic characteristics between the included and excluded subjects. Compared with the included subjects, the excluded subjects had lower education and income, higher BMI, a higher proportion of smokers and a history of depression. Therefore, there may be a serious selection bias in this study.

## 4 Discussion

### 4.1 Summary of main findings

This study investigated the longitudinal association between GDs and mental disorders in perimenopausal women by leveraging eight years of longitudinal follow-up data from the Swan Study. Our findings revealed significant associations between pelvic pain, pelvic prolapse/relaxation, and abnormal bleeding with symptoms of depression. Furthermore, pelvic pain and abnormal bleeding were significantly associated with insomnia, with insomnia playing a partial mediating role in the relationship between pelvic pain, abnormal bleeding, and depression. Importantly, this study pioneers the understanding of the longitudinal association between GDs in perimenopausal women and the progression of insomnia and depression, shedding light on the potential mediating role of insomnia.

### 4.2 Findings regarding depression

Our current research enhances and expands upon existing evidence in several key areas. Firstly, it demonstrates a significant association between pelvic pain, pelvic prolapse/relaxation, and abnormal bleeding in early perimenopausal women and symptoms of depression during mid- and late-menopause. These findings align with prior research indicating that women with depression often report higher pain levels, impaired physical function, and diminished quality of life compared to those without depression (23–25). For example, a nested case-control study by Arnold et al. (23), involving 425 participants, found that women with vulvodynia had a depression risk nearly three times higher than those without vulvodynia (*OR* = 2.99; 95% CI = 1.87-4.80). This result is significantly higher than the findings of the current study, possibly due to differences in study design and sample size. Notably, a systematic review and meta-analysis conducted by Trevor Thompson et al. revealed that individuals with depression may exhibit a lower pain threshold/tolerance to endogenous stimuli and are more prone to experiencing pain (26). Furthermore, pelvic pain can be a factor in the development of depression (24), which suggested that the two might share some common pathogenic mechanisms.

On the other hand, limited research has explored the association between menstrual problems and depression in postmenopausal women. One community-based epidemiological study, conducted as part of the Mental Health Study (MHS) within the SWAN ancillary study, reported findings that were not consistent with this study. Joyce T. Bromberger et al. found no significant association between current depression and abnormal bleeding patterns after adjusting for previous history of depression (10). Their study adopted a cross-sectional approach, whereas our focus lies in examining the longitudinal development pattern of depression. Nonetheless, our findings align with previous clinical observations, indicating that women with abnormal bleeding are more likely to experience psychological disturbances, including major depression, generalized anxiety, and compromised quality of life (27–29). Notably, in interviews conducted by Golden-Plotnik (27), a majority of the women attributed their symptoms of depression and anxiety to abnormal bleeding. Furthermore, Hae Nam Lee et al. (29) emphasized the underdiagnosis and undertreatment of depression and anxiety within the subgroup of women experiencing abnormal bleeding. They also found that the risk for depression in patients with abnormal uterine bleeding who had a history of minor surgery was high (*OR* = 2.96; 95% *CI* = 1.05-8.33). Our findings underscore the importance of comprehensive assessment and appropriate management strategies in clinical practice.

Previous studies on the association between pelvic floor dysfunction and depression have predominantly relied on cross-sectional surveys conducted among clinical populations (30–32). However, the association between pelvic prolapse/relaxation and depression has not been the main focus of these studies, and our study fills this research gap. The findings of this study indicate that the association between pelvic prolapse and depression may be stronger compared to that of pelvic pain and abnormal bleeding. Notably, prior studies have reported that up to 41% of women aged 50 to 79 exhibit some degree of pelvic organ prolapse (33). These findings underscore the importance of early diagnosis and intervention for pelvic prolapse/relaxation, as well as pelvic pain and abnormal bleeding, to mitigate the risk of physical and psychological complications in postmenopausal women.

### 4.3 Findings regarding insomnia

Our findings reveal a significant positive association between pelvic pain and the severity of insomnia, which is consistent with previous evidence (34, 35). For example, Horibe et al. (35) found that there was a significant association of sleep disturbance with persistent low back and pelvic pain (*OR*= 2.98; 95% *CI* = 1.31-6.75).These findings align with the insights provided by Kathleen T. Dunlap et al. (36), who found that women with pelvic pain were more likely to have an increase in rapid eye movement sleep onset latency and a decrease in rapid eye movement sleep, and also found that none of the women had obstructive sleep apnea. The severity of the sleep disorders may also need evidence from a larger sample of population studies. However, this also emphasizes the importance of the need for timely sleep interventions to prevent worsening of insomnia in the future. Similarly, our study’s findings regarding abnormal bleeding and insomnia closely align with those reported by Subhasmita Behera et al. (37). Notably, we identified insomnia as playing a partial mediating role in the association between pelvic pain, abnormal bleeding and depression. Consequently, incorporating interventions to enhance sleep quality among this specific patient population may contribute to reducing the risk of future depression.

However, this study did not find a significant association between pelvic prolapse/relaxation and insomnia, despite the consistent significant association between pelvic prolapse/relaxation and depression. There are two possible explanations for this. First, the measurement of insomnia in this study was based on methods reported in previous research, rather than using a validated and specific scale for insomnia, which could have introduced measurement error. Second, there is a certain degree of selection bias in the study, and the current sample may not be fully representative of the broader population for examining this particular relationship. Therefore, future research should consider using more reliable and validated tools for measuring insomnia, as well as addressing potential selection bias by ensuring a more diverse and representative sample. This could provide a more accurate understanding of the potential link between pelvic prolapse and insomnia, as well as help to clarify the underlying mechanisms.

### 4.4 Regarding clinical practice recommendations

In this study, we identified two distinct trajectory groups for both insomnia and depression: the high and low trajectory groups, characterized by stable levels over time, with symptoms consistently high or low from the beginning. These findings have important implications for clinical practice. First, the early identification of women in the high trajectory group can help healthcare providers recognize individuals at higher risk of persistent insomnia and depression. These patients may benefit from more intensive and comprehensive interventions targeting both conditions. Second, women in the low trajectory group, while at lower risk, could still benefit from regular monitoring to ensure symptoms remain under control without overburdening healthcare resources. The stability of these trajectories suggests that early, targeted care can effectively allocate resources by focusing on those most in need, potentially reducing long-term healthcare costs. Third, our findings emphasize the importance of incorporating routine insomnia screenings into clinical evaluations for patients with gynecological disorders, such as pelvic pain or abnormal uterine bleeding. Early detection of sleep disturbances in these patients may prevent the progression of depression, providing an opportunity for timely intervention and better long-term health management. Finally, although the GDs in this study are common among women, they may be associated with more serious underlying conditions, such as endometrial cancer or endometrial intraepithelial neoplasia (38). These conditions can, in turn, lead to the development of mental health disorders, including depression (39). Therefore, it is crucial for healthcare providers to consider a broad differential diagnosis when evaluating women presenting with symptoms such as abnormal bleeding. This approach not only ensures that more serious conditions are not overlooked but also helps in providing timely interventions for both the underlying medical condition and any associated mental health concerns.

### 4.5 Strengths and limitations

The study possesses several key strengths. First, it capitalizes on a relatively large sample size of women. Second, its longitudinal cohort design effectively mitigates recall bias. Third, the utilization of multiple and continuous repeated measures of insomnia and depression enables the identification of longitudinal patterns over time. However, it is crucial to acknowledge certain limitations within this study. Firstly, all information regarding GDs, as well as subsequent measurements of insomnia and depression during the follow-up period, relies on self-reporting, which may introduce inherent measurement errors. Secondly, in order to examine the longitudinal development of insomnia and depression over an extended duration, this study selected eight years of follow-up data, resulting in a reduced statistical power due to the exclusion of nearly half of the initial sample population. Thus, future research should aim to address this limitation by conducting a cohort study on a larger sample size. Third, the exclusion of study participants has introduced selection bias, which could potentially impact the results. Although the results from the inverse probability weighting analysis suggest robustness, caution is still needed in interpreting these findings. Fourth, the tool used to assess insomnia relies on general questions from the survey rather than a specifically developed and validated scale, which may introduce potential measurement errors. Fifth, this study only considered baseline GDs and did not account for the potential impact of changing GDs over time on insomnia and depression. Future studies could explore the effects of time-varying GDs using models such as linear mixed-effects models. Sixth, due to the design of the survey questions regarding GDs, GDs were treated as a binary variable in this study, preventing us from quantifying the relationship between the severity of GDs and insomnia and depression. Finally, this study recognized that some covariates, such as family income and menopausal stages, may vary over time and potentially interact with other variables in complex ways, which could introduce additional variability and potential bias. As a result, only baseline covariates were considered in this analysis. Given that previous studies have indicated a potential doubling of the co-risk of depression and pelvic pain among economically disadvantaged populations (40), the omission of the time-varying characteristics may somewhat impact the robustness of the study’s conclusions.

## 5 Conclusions

This study revealed significant associations between pelvic pain, pelvic prolapse/relaxation, and abnormal bleeding during early menopause, and depression during late menopause. Early detection and treatment interventions have the potential to reduce the risk of depression in later stages of menopause. Notably, insomnia partially mediates the link between pelvic pain, abnormal bleeding and depression, highlighting the importance of addressing sleep disorders in these individuals. Future research should aim to replicate the current findings by addressing existing limitations and expanding the sample size. Additionally, it is essential to conduct relevant animal studies to explore the underlying biological mechanisms in greater depth.

## Data Availability

The datasets presented in this study can be found in online repositories. The names of the repository/repositories and accession number(s) can be found below: https://www.swanstudy.org.
