# Supplementary material for: Association between gynecological disorders and insomnia and depression trajectories: a longitudinal study of middle-aged women
Source: Front Psychiatry. 2025 Jan 7;15:1515678. doi: 10.3389/fpsyt.2024.1515678 (PMC11747789; doi:10.3389/fpsyt.2024.1515678)
Supplement: Supplementary file 1 [file Table1.docx]

**Supplementary Materials**

**Association between gynecological disorders and insomnia and depression trajectories: a longitudinal study of middle-aged Women**

Huiyong Zhong ^1, *^, Fuling Zeng ^1^

1, Guang Zhou Baiyun District Maternal and Child Health Hospital, Guangzhou, 510410, China

* Correspondence to: Huiyong Zhong, Guang Zhou Baiyun District Maternal and Child Health Hospital, Guangzhou, 510410, China

E-mail addresses: 253168995@qq.com

**Supplementary Tables**

**Table S1.** Covariates missing information in the insomnia trajectory analysis sample

**Table S2.** Fit statistics of latent class mixture model for insomnia and depression

**Table S3**. Characteristics of study population by insomnia trajectory group

**Table S4**. Characteristics of study population by depression trajectory group

**Table S5**. Sensitivity analysis, association between gynecological disorders and insomnia trajectories among participants who completed all 5 waves of insomnia assessments (n=1817)

**Table S6**. Sensitivity analysis, association between gynecological disorders and insomnia trajectories based on weighted population

**Table S7**. Sensitivity analysis, association between gynecological disorders and depression trajectories among participants who completed all 5 waves of depression assessments (n=1050)

**Table S8**. Sensitivity analysis, association between gynecological disorders and depression trajectories based on weighted population

**Table S9**. Sensitivity analysis, associations between gynecological disorders, insomnia and depression trajectories with additional adjustment for history of hormone replacement therapy

**Table S10**. Sensitivity analysis, the mediating effect of insomnia in the association between gynecological diseases and depression trajectories.

**Table S11**. Characteristics between included and excluded study population.

**Table S1.** Covariates missing information in the insomnia trajectory analysis sample

| Covariates | Missing number | Missing proportion (%) |
| --- | --- | --- |
| Age | 2 | <0.001 |
| BMI | 71 | 0.032 |
| Marital status | 2 | <0.001 |
| Education | 15 | 0.007 |
| Family income | 91 | 0.041 |
| History of depression | 92 | 0.041 |
| Smoking | 2 | <0.001 |

**Table S2.** Fit statistics of latent growth mixture model for insomnia and depression.

| Outcome | Number of classes estimated | AIC | BIC | Relative entropy | Class membership (%) | Mean posterior probabilities |
| --- | --- | --- | --- | --- | --- | --- |
| Insomnia | 1 | 50314.07 | 50348.30 | 1.00 | Class 1 = 100.00 | Class 1 = 1.00 |
|  | 2 | 49834.77 | 49891.81 | 0.54 | Class 1 = 49.26 Class 2 = 50.74 | Class 1 = 0.82 Class 2 = 0.91 |
|  | 3 | 49712.51 | 49792.37 | 0.54 | Class 1 = 30.04 Class 2 = 35.45 Class 3 = 34.51 | Class 1 = 0.87 Class 2 = 0.82 Class 3 = 0.67 |
|  | 4 | 49702.99 | 49805.66 | 0.53 | Class 1 = 17.00 Class 2 = 37.21 Class 3 = 17.82 Class 4 = 27.97 | Class 1 = 0.56 Class 2 = 0.83 Class 3 = 0.80 Class 4 = 0.64 |
|  | 5 | 49678.99 | 49804.47 | 0.65 | Class 1 = 36.40 Class 2 = 4.92 Class 3 = 9.02 Class 4 = 28.06 Class 5 = 21.60 | Class 1 = 0.85 Class 2 = 0.70 Class 3 = 0.86 Class 4 = 0.67 Class5 = 0.75 |
| Depression | 1 | 54243.40 | 54276.26 | 1.00 | Class 1 = 100.00 | Class 1 = 1.00 |
|  | 2 | 53309.36 | 53364.13 | 0.64 | Class 1 = 67.23 Class 2 = 32.77 | Class 1 = 0.90 Class 2 = 0.93 |
|  | 3 | 53125.37 | 53202.05 | 0.64 | Class 1 = 56.87 Class 2 = 15.17 Class 3 = 27.96 | Class 1 = 0.89 Class 2 = 0.88 Class 3 = 0.74 |
|  | 4 | 53087.30 | 53185.89 | 0.68 | Class 1 = 4.24 Class 2 = 54.50 Class 3 = 19.53 Class 4 = 21.73 | Class 1 = 0.87 Class 2 = 0.89 Class 3 = 0.63 Class 4 = 0.80 |
|  | 5 | 53105.86 | 53226.36 | 0.60 | Class 1 = 9.0 Class 2 = 56.99 Class 3 = 13.81 Class 4 = 0 Class 5 = 20.20 | Class 1 = 0.83 Class 2 = 0.86 Class 3 = 0.44 Class 4 = - Class 5 = 0.64 |

**Table S3**. Characteristics of study population by insomnia trajectory group

| Variate | n | Class 1  (n = 1092) | Class 2  (n =1125) | *t*/*χ^2^* |
| --- | --- | --- | --- | --- |
| Age | - | 45.83±2.649 | 46.05±2.671 | -1.907 |
| BMI | - | 27.31±6.689 | 29.07±7.608 | -5.790*** |
| Race |  |  |  | 45.545*** |
| Black, African American | 540 (24.4) | 251 (23.0) | 289 (25.7) |  |
| Chinese or Chinese American | 213 (9.6) | 121 (11.1) | 92 (8.2) |  |
| Japanese or Japanese American | 249 (11.2) | 163 (14.9) | 86 (7.6) |  |
| Caucasian/White non-Hispanic | 1091 (49.2) | 486 (44.5) | 605 (53.8) |  |
| Hispanic | 124 (5.6) | 71 (6.5) | 53 (4.7) |  |
| Marital status |  |  |  | 6.566 |
| Single/never married | 275 (12.4) | 143 (13.1) | 132 (11.7) |  |
| Currently married or living as married | 1486 (67.0) | 743 (68.0) | 743 (66.0) |  |
| Separated | 85 (3.8) | 32 (2.9) | 53 (4.7) |  |
| Widowed | 53 (2.4) | 25 (2.3) | 28 (2.5) |  |
| Divorced | 318 (14.3) | 149 (13.6) | 169 (15.0) |  |
| Menopausal Status |  |  |  | 31.695*** |
| Post by bilateral salpingo oophorectomy | 31 (1.4) | 8(0.7) | 23(2.0) |  |
| Natural post | 108 (4.9) | 60(5.5) | 48(4.3) |  |
| Late perimenopause | 166(7.5) | 68(6.2) | 98(8.7) |  |
| Early perimenopause | 1284(57.9) | 633(58.0) | 651(57.9) |  |
| Premenopausal | 378(17.1) | 220(20.1) | 158(14.0) |  |
| Unknown due to hormones use | 250(11.3) | 103(9.4) | 147(13.1) |  |
| Education |  |  |  | 6.336 |
| Less than High School | 119 (5.4) | 55 (5.0) | 64 (5.7) |  |
| High school only | 357 (16.1) | 169 (15.5) | 188 (16.7) |  |
| Some College/Technical School | 698 (31.5) | 325 (29.8) | 373 (33.2) |  |
| College degree | 486 (21.9) | 255 (23.4) | 231 (20.5) |  |
| Post Graduate Education | 557 (25.1) | 288 (26.4) | 269 (23.9) |  |
| Family income |  |  |  | 24.659*** |
| Less than $19, 999 | 227 (10.2) | 82 (7.5) | 145 (12.9) |  |
| $20, 000 to $49, 999 | 634 (28.6) | 295 (27.0) | 339 (30.1) |  |
| $50, 000 to $99, 999 | 900 (40.6) | 468 (42.9) | 432 (38.4) |  |
| $100,000 to $ more | 456 (20.6) | 247 (22.6) | 209 (18.6) |  |
| Smoking |  |  |  | 3.239 |
| No | 1907 (86.0) | 954 (87.4) | 953 (84.7) |  |
| Yes | 310 (14.0) | 138 (12.6) | 172 (15.3) |  |
| History of depression |  |  |  | 84.042*** |
| No | 1578 (71.2) | 875 (80.1) | 703 (62.5) |  |
| Yes | 639 (28.8) | 217 (19.9) | 422 (37.5) |  |
| History of hormone replacement therapy |  |  |  | 6.286* |
| No | 1980 (89.3) | 994 (91.0) | 986 (87.6) |  |
| Yes | 237 (10.7) | 98 (9.0) | 139 (12.4) |  |
| Pelvic pain |  |  |  | 36.011*** |
| No | 1876 (84.6) | 975 (89.3) | 901 (80.1) |  |
| Yes | 341 (15.4) | 117 (10.7) | 224 (19.9) |  |
| Pelvic prolapse or relaxation |  |  |  | 2.036 |
| No | 2155 (97.2) | 1067 (97.7) | 1088 (96.7) |  |
| Yes | 62 (2.8) | 25 (2.3) | 37 (3.3) |  |
| Abnormal bleeding |  |  |  | 16.514*** |
| No | 1877 (84.7) | 959 (87.8) | 918 (81.6) |  |
| Yes | 340 (15.3) | 133 (12.2) | 207 (18.4) |  |
| Number of GDs |  |  |  | 40.126*** |
| 0 | 1617 (72.9) | 857 (78.5) | 760 (67.6) |  |
| 1 | 467 (21.1) | 196 (17.9) | 271 (17.9) |  |
| ≥2 | 133 (6.0) | 39 (3.6) | 94 (3.6) |  |
| Note: ***, *P*<0.001 | | | | |

**Table S4**. Characteristics of study population by depression trajectory group.

| Variate | n | Class 1  (n = 1188) | Class 2  (n =579) | *t*/*χ^2^* |
| --- | --- | --- | --- | --- |
| Age | - | 46.01 ± 2.639 | 45.77 ± 2.633 | 1.820 |
| BMI | - | 27.38 ± 6.709 | 29.01 ± 8.049 | -4.486*** |
| Race |  |  |  | 10.462* |
| Black, African American | 439 (24.8) | 275 (23.1) | 164 (28.3) |  |
| Chinese or Chinese American | 185 (10.5) | 123 (10.4) | 62 10.7) |  |
| Japanese or Japanese American | 232 (13.1) | 147 (12.4) | 85 (14.7) |  |
| Caucasian/White non-Hispanic | 911 (51.6) | 643 (54.1) | 268 (46.3) |  |
| Marital status |  |  |  | 31.642*** |
| Single/never married | 210 (11.9) | 124 (10.4) | 86 (14.9) |  |
| Currently married or living as married | 1213 (68.6) | 859 (72.3) | 354 (61.1) |  |
| Separated | 56 (3.2) | 25 (2.1) | 31 (5.4) |  |
| Widowed | 42 (2.4) | 22 (1.9) | 20 (3.5) |  |
| Divorced | 246 (13.9) | 158 (13.3) | 88 (15.2) |  |
| Menopausal Status |  |  |  | 9.999 |
| Post by bilateral salpingo oophorectomy | 26 (1.5) | 14 (1.2) | 12 (2.1) |  |
| Natural post | 72 (4.1) | 42 (3.5) | 30 (5.2) |  |
| Late perimenopause | 133 (7.5) | 95 (8.0) | 38 (6.6) |  |
| Early perimenopause | 1006 (56.9) | 665 (56.0) | 341 (58.9) |  |
| Premenopausal | 314 (17.8) | 227 (19.1) | 87 (15.0) |  |
| Unknown due to hormones use | 216 (12.2) | 145 (12.2) | 71 (12.3) |  |
| Education |  |  |  | 18.226*** |
| Less than High School | 47 (2.7) | 21 (1.8) | 26 (4.5) |  |
| High school only | 262 (14.8) | 169 (14.2) | 93 (16.1) |  |
| Some College/Technical School | 570 (32.3) | 381 (32.1) | 189 (32.6) |  |
| College degree | 414 (23.4) | 273 (23.0) | 141 (24.4) |  |
| Post Graduate Education | 474 (26.8) | 344 (29.0) | 130 (22.5) |  |
| Family income |  |  |  | 77.657*** |
| Less than $19, 999 | 126 (7.1) | 50 (4.7) | 76 (13.1) |  |
| $20, 000 to $49, 999 | 501 (28.4) | 303 (25.5) | 198 (34.2) |  |
| $50, 000 to $99, 999 | 751 (42.5) | 533 (44.9) | 218 (37.7) |  |
| $100,000 to $ more | 389 (22.0) | 302 (25.4) | 87 (15.0) |  |
| Smoking |  |  |  | 16.857*** |
| No | 1536 (86.9) | 1060 (89.2) | 476 (82.2) |  |
| Yes | 231 (13.1) | 128 (10.8) | 103 (17.8) |  |
| History of depression |  |  |  | 225.506*** |
| No | 1304 (73.8) | 1007 (84.8) | 297 (51.3) |  |
| Yes | 463 (26.2) | 181 (15.2) | 282 (48.7) |  |
| History of hormone replacement therapy |  |  |  | 0.024 |
| No | 1564 (88.5) | 1053 (88.6) | 511 (88.3) |  |
| Yes | 203 (11.5) | 135 (11.4) | 68 (11.7) |  |
| Pelvic pain |  |  |  | 15.569*** |
| No | 1501 (84.9) | 1037 (87.3) | 464 (80.1) |  |
| Yes | 266 (15.1) | 151 (12.7) | 115 (19.9) |  |
| Pelvic prolapse or relaxation |  |  |  | 6.651** |
| No | 1719 (97.3) | 1164 (98.0) | 555 (95.8) |  |
| Yes | 48 (2.7) | 24 (2.0) | 24 (4.1) |  |
| Abnormal bleeding |  |  |  | 9.436** |
| No | 1495 (84.6) | 1027 (86.4) | 468 (80.8) |  |
| Yes | 272 (15.4) | 161 (13.6) | 111 (19.2) |  |
| Number of GDs |  |  |  | 22.129*** |
| 0 | 1293 (73.2) | 905 (76.2) | 388 (67.0) |  |
| 1 | 372 (21.1) | 232 (19.5) | 140 (24.2) |  |
| ≥2 | 102 (5.8) | 51 (4.3) | 51 (8.8) |  |
| Note: *, *P*<0.05; **, *P*<0.01; ***, *P*<0.001 | | | | |

**Table S5**. Sensitivity analysis, association between gynecological disorders and insomnia trajectories among participants who completed all 5 waves of insomnia assessments (n=1817)

| Gynecological disorders | Crude model | |  | Adjusted Model | |
| --- | --- | --- | --- | --- | --- |
|  | *OR* (95% *CI*) | *P* |  | *OR* (95% *CI*) | *P* |
| Pelvic pain |  |  |  |  |  |
| No | 1.00 (Ref.) |  |  | 1.00 (Ref.) |  |
| Yes | 1.91 (1.47-2.49) | <0.001 |  | 1.70 (1.29-2.24) | <0.001 |
| Pelvic prolapse or relaxation |  |  |  |  |  |
| No | 1.00 (Ref.) |  |  | 1.00 (Ref.) |  |
| Yes | 1.25 (0.70-2.23) | 0.459 |  | 1.02 (0.55-1.89) | 0.941 |
| Abnormal bleeding |  |  |  |  |  |
| No | 1.00 (Ref.) |  |  | 1.00 (Ref.) |  |
| Yes | 1.57 (1.21-2.04) | <0.001 |  | 1.41 (1.07-1.85) | 0.016 |
| Number of GDs |  |  |  |  |  |
| 0 | 1.00 (Ref.) |  |  | 1.00 (Ref.) |  |
| 1 | 1.51 (1.20-1.89) | <0.001 |  | 1.44 (1.13-1.83) | 0.003 |
| ≥2 | 2.36 (1.55-3.58) | <0.001 |  | 1.82 (1.17-2.83) | 0.008 |
| *P* for trend | - | <0.001 |  | - | <0.001 |

Note: “Low” as reference trajectory; Crude model: no adjustment; Adjusted model: adjusted for age, BMI, race, marital status, menopausal status, education, family income, smoking, and depression history.

**Table S6**. Sensitivity analysis, association between gynecological disorders and insomnia trajectories based on weighted population

| Gynecological disorders | Crude model | |  | Adjusted Model | |
| --- | --- | --- | --- | --- | --- |
|  | *OR* (95% *CI*) | *P* |  | *OR* (95% *CI*) | *P* |
| Pelvic pain |  |  |  |  |  |
| No | 1.00 (Ref.) |  |  | 1.00 (Ref.) |  |
| Yes | 2.07 (1.63-2.64) | <0.001 |  | 1.82 (1.41-2.36) | <0.001 |
| Pelvic prolapse or relaxation |  |  |  |  |  |
| No | 1.00 (Ref.) |  |  | 1.00 (Ref.) |  |
| Yes | 1.45 (0.87-2.43) | 0.156 |  | 1.21 (0.71-2.07) | 0.480 |
| Abnormal bleeding |  |  |  |  |  |
| No | 1.00 (Ref.) |  |  | 1.00 (Ref.) |  |
| Yes | 1.63 (1.28-2.06) | <0.001 |  | 1.42 (1.11-1.82) | 0.006 |
| Number of GDs |  |  |  |  |  |
| 0 | 1.00 (Ref.) |  |  | 1.00 (Ref.) |  |
| 1 | 1.56 (1.27-1.92) | <0.001 |  | 1.47 (1.18-1.83) | <0.001 |
| ≥2 | 2.72 (1.85-4.00) | <0.001 |  | 2.04 (1.36-3.07) | <0.001 |
| *P* for trend | - | <0.001 |  | - | <0.001 |

Note: “Low” as reference trajectory; Crude model: no adjustment; Adjusted model: adjusted for age, BMI, race, marital status, menopausal status, education, family income, smoking, and depression history.

**Table S7**. Sensitivity analysis, association between gynecological disorders and depression trajectories among participants who completed all 5 waves of depression assessments (n=1050)

| Gynecological disorders | Crude model | |  | Adjusted Model | |
| --- | --- | --- | --- | --- | --- |
|  | *OR* (95%CI) | *P* |  | *OR* (95%CI) | *P* |
| Pelvic pain |  |  |  |  |  |
| No | 1.00 (Ref.) |  |  | 1.00 (Ref.) |  |
| Yes | 1.69 (1.20-2.36) | 0.003 |  | 1.46 (1.00-2.23) | 0.050 |
| Pelvic prolapse or relaxation |  |  |  |  |  |
| No | 1.00 (Ref.) |  |  | 1.00 (Ref.) |  |
| Yes | 1.56 (0.71-3.44) | 0.267 |  | 1.48 (0.61-3.61) | 0.391 |
| Abnormal bleeding |  |  |  |  |  |
| No | 1.00 (Ref.) |  |  | 1.00 (Ref.) |  |
| Yes | 1.43 (1.01-2.02) | 0.042 |  | 1.42 (0.96-2.10) | 0.082 |
| Number of GDs |  |  |  |  |  |
| 0 | 1.00 (Ref.) |  |  | 1.00 (Ref.) |  |
| 1 | 1.41 (1.04-1.91) | 0.029 |  | 1.56 (1.11-2.20) | 0.011 |
| ≥2 | 2.08 (1.21-3.56) | 0.008 |  | 1.47 (0.80-2.69) | 0.215 |
| *P* for trend | - | 0.001 |  | - | 0.017 |

Note: “Low” as reference trajectory; Crude model: no adjustment; Adjusted model: adjusted for age, BMI, race, marital status, menopausal status, education, family income, smoking, and depression history.

**Table S8**. Sensitivity analysis, association between gynecological disorders and depression trajectories based on weighted population

| Gynecological disorders | Crude model | |  | Adjusted Model | |
| --- | --- | --- | --- | --- | --- |
|  | *OR* (95%CI) | *P* |  | *OR* (95%CI) | *P* |
| Pelvic pain |  |  |  |  |  |
| No | 1.00 (Ref.) |  |  | 1.00 (Ref.) |  |
| Yes | 1.70 (1.30-2.22) | <0.001 |  | 1.47 (1.09-1.99) | 0.011 |
| Pelvic prolapse or relaxation |  |  |  |  |  |
| No | 1.00 (Ref.) |  |  | 1.00 (Ref.) |  |
| Yes | 2.10 (1.18-3.73) | 0.011 |  | 1.81 (0.96-3.40) | 0.067 |
| Abnormal bleeding |  |  |  |  |  |
| No | 1.00 (Ref.) |  |  | 1.00 (Ref.) |  |
| Yes | 1.51 (1.16-1.97) | 0.002 |  | 1.39 (1.03-1.87) | 0.033 |
| Number of GDs |  |  |  |  |  |
| 0 | 1.00 (Ref.) |  |  | 1.00 (Ref.) |  |
| 1 | 1.41 (1.11-1.79) | 0.006 |  | 1.44 (1.09-1.89) | 0.009 |
| ≥2 | 2.33 (1.55-3.50) | <0.001 |  | 1.72 (1.10-2.68) | 0.018 |
| *P* for trend | - | <0.001 |  | - | 0.001 |

Note: “Low” as reference trajectory; Crude model: no adjustment; Adjusted model: adjusted for age, BMI, race, marital status, menopausal status, education, family income, smoking, and depression history.

**Table S9**. Sensitivity analysis, associations between gynecological disorders, insomnia and depression trajectories with additional adjustment for history of hormone replacement therapy

| Gynecological disorders | insomnia trajectories | |  | depression trajectories | |
| --- | --- | --- | --- | --- | --- |
|  | *OR* (95%CI) | *P* |  | *OR* (95%CI) | *P* |
| Pelvic pain |  |  |  |  |  |
| No | 1.00 (Ref.) |  |  | 1.00 (Ref.) |  |
| Yes | 1.88 (1.46-2.43) | <0.001 |  | 1.45 (1.07-1.95) | 0.015 |
| Pelvic prolapse or relaxation |  |  |  |  |  |
| No | 1.00 (Ref.) |  |  | 1.00 (Ref.) |  |
| Yes | 1.19 (0.69-2.05) | 0.536 |  | 1.87 (0.97-3.59) | 0.060 |
| Abnormal bleeding |  |  |  |  |  |
| No | 1.00 (Ref.) |  |  | 1.00 (Ref.) |  |
| Yes | 1.43 (1.11-1.84) | 0.006 |  | 1.48 (1.09-2.01) | 0.011 |
| Number of GDs |  |  |  |  |  |
| 0 | 1.00 (Ref.) |  |  | 1.00 (Ref.) |  |
| 1 | 1.51 (1.21-1.88) | <0.001 |  | 1.51 (1.15-1.98) | 0.003 |
| ≥2 | 2.08 (1.39-3.16) | <0.001 |  | 1.75 (1.10-2.79) | 0.018 |
| *P* for trend | - | <0.001 |  | - | <0.001 |

Note: “Low” as reference trajectory; these models adjusted for age, BMI, race, marital status, menopausal status, education, family income, smoking, depression history, and history of hormone replacement therapy.

**Table S10**. Sensitivity analysis, the mediating effect of insomnia in the association between gynecological diseases and depression trajectories.

| Gynecological disorders | Average ACME  *OR* (95% *CI*) | Average ADE  *OR* (95% *CI*) | Total effect  *OR* (95% *CI*) | Proportion mediated (%) |
| --- | --- | --- | --- | --- |
| Pelvic pain | 1.018 (1.005-1.030) | 1.056 (1.000-1.128) | 1.075 (1.014-1.150) | 23.9 |
| Abnormal bleeding | 1.010 (1.000-1.020) | 1.067 (1.006-1.139) | 1.078 (1.016-1.150) | 14.3 |
| Number of GDs | 1.010 (1.000-1.020) | 1.073 (1.017-1.128) | 1.084 (1.025-1.139) | 11.8 |
| Note: ACME, average causal mediation effect; ADE, average direct effect. | | | | |

**Table S11**. Characteristics between included and excluded study population.

| Variate | n | Included  (n = 1767) | Excluded  (n = 895) | *t*/*χ^2^* |
| --- | --- | --- | --- | --- |
| Age | 2662 | 45.93 ± 2.64 | 45.87± 2.77 | 0.530 |
| BMI | 2662 | 27.94 ± 7.20 | 29.53 ± 7.18 | -5.405*** |
| Race |  |  |  | 428.98*** |
| Black, African American | 702 (26.4) | 439 (24.8) | 263 (29.4) |  |
| Chinese or Chinese American | 230 (8.6) | 185 (10.5) | 45 (5.0) |  |
| Japanese or Japanese American | 262 (9.8) | 232 (13.1) | 30 (3.4) |  |
| Caucasian/White non-Hispanic | 1297 (48.7) | 911 (51.6) | 386 (43.1) |  |
| Hispanic | 171 (6.4) | 0 (0.0) | 171 (19.1) |  |
| Marital status |  |  |  | 21.390*** |
| Single/never married | 338 (12.7) | 210 (11.9) | 128 (14.3) |  |
| Currently married or living as married | 1754 (65.9) | 1213 (68.6) | 541 (60.4) |  |
| Separated | 106 (4.0) | 56 (3.2) | 50 (5.6) |  |
| Widowed | 67 (2.5) | 42 (2.4) | 25 (2.8) |  |
| Divorced | 397 (14.9) | 246 (13.9) | 151 (16.9) |  |
| Menopausal Status |  |  |  | 67.036*** |
| Post by bilateral salpingo oophorectomy | 39 (1.5) | 26 (1.5) | 13 (1.5) |  |
| Natural post | 139 (5.2) | 72 (4.1) | 67 (7.5) |  |
| Late perimenopause | 201 (7.6) | 133 (7.5) | 68 (7.6) |  |
| Early perimenopause | 1524 (57.3) | 1006 (56.9) | 518 (57.9) |  |
| Premenopausal | 440 (16.5) | 314 (17.8) | 126 (14.1) |  |
| Pregnant/breastfeeding | 2 (0.1) | 0 (0.0) | 2 (0.2) |  |
| Unknown due to hormones use | 297 (11.2) | 216 (12.2) | 81 (9.1) |  |
| Unknown due to hysterectomy | 20 (0.8) | 0 (0.0) | 20 (2.2) |  |
| Education |  |  |  | 132.12*** |
| Less than High School | 157 (5.9) | 46 (2.6) | 111 (12.4) |  |
| High school only | 448 (16.8) | 263 (14.9) | 185 (20.7) |  |
| Some College/Technical School | 838 (31.5) | 571 (32.3) | 267 (29.8) |  |
| College degree | 566 (21.3) | 415 (23.5) | 151 (16.9) |  |
| Post Graduate Education | 653 (24.5) | 472 (26.7) | 181 (20.2) |  |
| Family income |  |  |  | 94.485*** |
| Less than $19, 999 | 298 (11.2) | 128 (7.2) | 170 (19.0) |  |
| $20, 000 to $49, 999 | 773 (29.0) | 499 (28.2) | 274 (30.6) |  |
| $50, 000 to $99, 999 | 1054 (39.6) | 751 (42.5) | 303 (33.9) |  |
| $100,000 to $ more | 537 (20.2) | 389 (22.0) | 148 (16.5) |  |
| Smoking |  |  |  | 21.878*** |
| No | 2252 (84.6) | 1536 (86.9) | 716 (80.0) |  |
| Yes | 410 (15.4) | 231 (13.1) | 179 (20.0) |  |
| History of depression |  |  |  | 37.1*** |
| No | 1851 (69.5) | 1297 (73.4) | 554 (61.9) |  |
| Yes | 811 (30.5) | 470 (26.6) | 341 (38.1) |  |
| Pelvic pain |  |  |  | 0.302 |
| No | 2254 (84.7) | 1501 (84.9) | 753 (84.1) |  |
| Yes | 408 (15.3) | 266 (15.1) | 142 (15.9) |  |
| Pelvic prolapse or relaxation |  |  |  | 1.897 |
| No | 2581 (97.0) | 1719 (97.3) | 862 (96.3) |  |
| Yes | 81 (3.0) | 48 (2.7) | 33 (3.7) |  |
| Abnormal bleeding |  |  |  | 0.018 |
| No | 2254 (84.7) | 1495 (84.6) | 759 (84.8) |  |
| Yes | 408 (15.3) | 272 (15.4) | 136 (15.2) |  |
| Number of GDs |  |  |  | 1.376 |
| 0 | 1941 (72.9) | 1293 (73.2) | 648 (72.4) |  |
| 1 | 557 (20.9) | 372 (21.1) | 185 (20.7) |  |
| ≥2 | 164 (6.2) | 102 (5.8) | 62 (6.9) |  |
| Note: The 2662 subjects were those remaining after excluding missing information on pelvic pain, pelvic prolapse or relaxation, and abnormal bleeding. Comparisons of differences between groups were made after filling in the missing data. *, *P*<0.05; **, *P*<0.01; ***, *P*<0.001 | | | | |
